# Metastases to the kidney from primary lung cancer : clinicopathological analysis of six cases in a single center

**DOI:** 10.1186/s13000-023-01344-6

**Published:** 2023-05-09

**Authors:** Hui Lian, Xinyu Pan, Bo Hong, Jie Min, Fengbo Huang

**Affiliations:** 1 Linping Campus, The Second Affiliated Hospital of Zhejiang University School of Medicine, Hangzhou, China; 2 Hangzhou City Linping District Maternal and Child Care Hospital, Hangzhou, China; 3 The Department of Pathology, Second Affiliated Hospital of Zhejiang University School of Medicine, Hangzhou, China; 4 The Department of Radiology, Second Affiliated Hospital of Zhejiang University School of Medicine, Hangzhou, China; 5 Key Laboratory of Tumor Microenvironment and Immune Therapy of Zhejiang Province, Hangzhou, China

**Keywords:** Kidney, Metastasis, Lung Cancer, Clinicopathological features, Diagnosis, Treatment, Prognosis

## Abstract

**Objectives:**

Cancer metastasis to the kidney is a rare event. We retrospectively analyzed clinicopathologic characteristics in 6 cases of diagnosed renal metastases from primary lung cancer. We also provide clinical follow-up data and brief review of the literature.

**Methods:**

Immunohistochemistry was used to evaluate the expression of TTF-1, NapsinA, CK7, CK(AE1/AE3), P63, P40, CgA, PAX-8, GATA3 and Ki-67 in primary tumor and metastases. Additionally, the clinical characteristics, imaging features, diagnosis, and treatment were analyzed.

**Results:**

With the help of immunohistochemistry and combined clinical history, we found four cases were lung adenocarcinomas, one case was lung squamous cell carcinoma, and the other case was lung small cell carcinoma metastases to the kidney.The patients were all male by gender and had a mean age of 62 years, and metastasis to the left kidney were more universal. Most of the tumors histological grade originating from the lung were poorly-moderately differentiated, and the time to metastasis to the kidney was relatively short for squamous lung cancer and small cell lung cancer, while the time to metastasis for lung adenocarcinoma was related to its degree of differentiation. Overall, we found the prognosis of lung cancer patients with renal metastases were poor especially with multi-site metastases.

**Conclusions:**

Distinguishing primary and secondary tumors of the kidney is essential to guide treatment and prevent unnecessary surgery, so clinical information, radiology, histological correlation of the primary tumor, and immunohistochemical findings help the pathologist determine correct diagnosis.

## Introduction

The kidney was considered to be an organ where tumours rarely metastasized.Through autopsy, the kidney was found which was only the 12th most common organ involved with metastatic disease,with an incidence of 12.6% [1]. Renal metastases are rare, however, the most primary focus is the lung according to the literature [2]. Most studies on renal metastases from lung were case reports, and the clinical and pathological features of lung cancer metastases to the kidneys were not further investigated.

Lung cancers are traditionally classified as small cell (SCLC) or non-small cell (NSCLC), SCLCs are malignant tumors that account for approximately 15% of lung cancers, then NSCLCs account for about 85% of all lung cancers and include any type of lung cancer other than SCLCs [3, 4]. The leading cause of deaths in lung cancer has been attributed to metastatic involvement of distant organs. The common sites for lung cancer metastases include the bone, brain, liver, and adrenal glands, while isolated kidney involvement by primary lung cancer is a rare entity [5]. Prior studies have demonstrated that based on the histological subtype, sites of tumor metastasis may vary [6].

In this study, we analyzed 6 cases of metastases to the kidney from different histological subtype of primary lung cancer including lung adenocarcinoma, lung squamous cell carcinoma and lung small cell carcinoma at the Second Affiliated Hospital of Zhejiang University School of Medicine, and tried to describe their clinical features, clinicopathological features, treatment, and prognosis.

## Materials and methods

### Case selection

We found seven cases of metastases to the kidney from primary lung cancer between January 2010 to January 2022 in the archives of our department of pathology, and one case was excluded due to incomplete clinical information.The inclusion criteria were as follows: malignant tumors other than kidney were pathologically confirmed, renal metastases were pathologically diagnosed following puncture biopsy or surgical resection, and the source of renal metastases was consistent with the primary lesion. Clinical data of the cases were collected, including demographic information, time of diagnosis of the primary lesion, time of onset of renal metastases (time of imaging findings), symptoms, metastases from other sites, puncture pathology, and surgery. Biopsy or surgical specimens were evaluated by the pathologist and immunohistochemical staining was performed to determine the diagnosis. Six of the included cases were followed up to document their survival. Overall survival (OS) of patients was defined as the time from the diagnosis of the primary lesion to the last follow-up visit or death.The TNM staging involved in this study refers to the 2017 TNM classification of malignant tumours, eight edition.

### Immunohistochemistry

The tissues were fixed in formalin, dehydrated, and embedded in paraffin, after which 4-µm thin sections were cut. Immunohistochemical staining was performed using the En Vension method, with heating in a rice cooker to repair the antigen. We purchased commercially available antibodies for the following antigens: TTF-1, NapsinA, CK7, CK(AE1/AE3), P63, P40,CgA,Syn,PAX-8,GATA3 and Ki-67(Beijing Zhongshan Jinqiao Biotechnology Co., Ltd).

## Results

### Clinical features of patients

**Fig. 1:**
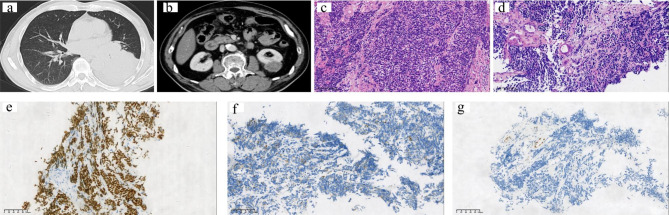
Renal Metastasis from Primary Lung Small Cell Carcinoma from Case 3 **(a)** Computed tomography (CT) scan showing the left lobe nodule. **(b)** CT scan showing the isolated left kidney metastasis. **(c)** The tumor cells were from lung (H&E, ×200). (d)The tumor cells were from kidney (H&E, ×200). Immunohistochemical studies revealed that the tumor cells were positive for TTF-1 (e, ×200) and CgA(f, ×200), but GATA3 was negative (g, ×200)

**Fig. 2:**
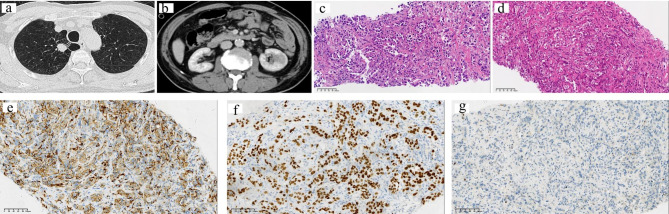
Renal Metastasis from Primary Lung Squamous Cell Carcinoma from Case 4 **(a)** CT scan showing the right lobe nodule. **(b)** CT scan showing the isolated left kidney metastasis. **(c)** The tumor cells were from lung (H&E, ×200). (d) The tumor cells were from kidney (H&E, ×200). Immunohistochemical studies revealed that the tumor cells were positive for P40(e, ×200) and P63(f, ×200), but GATA3 was negative (g, ×200)

**Fig. 3:**
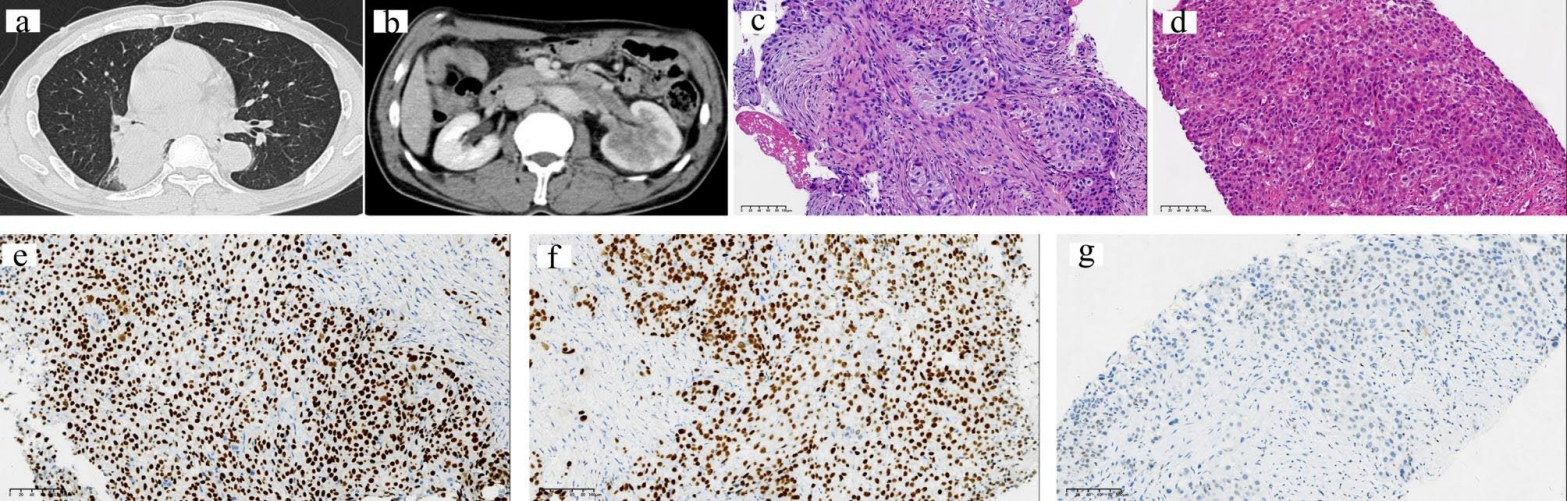
Renal Metastasis from Primary Lung Adenocarcinoma from Case 5 **(a)** CT scan showing the right lobe nodule; **(b)** CT scan showing the isolated left kidney metastasis; **(c)** The tumor cells were from lung (H&E, ×200). **(d)** The tumor cells were from kidney (H&E, ×200). Immunohistochemical studies revealed that the tumor cells were positive for NapsinA(e, ×200) and TTF-1(f, ×200), but PAX-8 was negative (g, ×200)

All 6 patients were male by gender, with an average age of 62 years, including 3 cases of ipsilateral renal metastases and 3 cases of contralateral renal metastases, with more metastases in the left kidney (n = 4) (Table 1). Case 3 showed that the left lung cancer with metastasis to the left kidney (Fig. 1a,b), and cases 4 with cases 5 showed that the right lung cancer with metastasis to the left kidney (Figs. 2a and b and 3a and b). In general, the average size of the tumor was 3.9 cm. Due to the different histological types of lung tumors, the time to metastasis varied widely, with a mean time to metastasis of 15 months and only 2 cases presented with clinical symptoms of back pain and hematuria. There were 4 cases of isolated metastasis of lung cancer to the kidney, and only 2 cases metastasized to other sites, and all of them metastasized to the brain (Table 1) .Three of the patients underwent nephrectomy, one with laparoscopic radical nephrectomy and two with laparoscopic partial nephrectomy in our manuscript. They were not found to have metastases elsewhere before undergoing nephrectomy, and the reasons for surgery in each of the three patients were therapeutic purpose, suspected primary tumor of kidney and poor kidney function.

**Table 1:** Clinical characteristics of the Six Metastases to the Kindney from Primary Lung Cancer Patients

sr No.	Age/Sex	Primary organ site (lung)	Initial clinical treatment	Metastasis organ site (renal)	Diameter of largest renal mass (cm)	Time to renal metastasis (months)	Symptoms	Other site metastasis	Surgery (Reason for surgery)	Clinical therapy	OS (months)	Outcome at last follow-up
1	64/F	L	CT	R	6	12	N	Y(brain)	Y(Curative intent)	CT and RT	14	DOD
2	55/F	L	Right lobe resection	L	2.5	36	N	N	Y(Suspected renal primary tumor)	CT	55	FU
3	64/F	L	CT	L	2.6	4	N	Y(brain)	N	CT and RT	15	DOD
4	56/F	R	CT and IT	L	6.3	7	Y	N	N	CT and IT	7	DOD
5	68/F	R	CT and IT	L	2.7	7	N	N	N	CT and IT	24	FU
6	66/F	R	Right lobe resection	R	3.3	24	Y	N	Y(Poorly functioning kidney)	CT and IT	16	FU

M, male; F, female; R, right; L, left;N,no;Y,yes;RN, radical nephrectomy; CT, chemotherapy; RT, radiotherapy; IT,immunotherapy;FU, follow-up; DOD, dead of disease ; OS: overall survival

### Pathologic and immunophenotypic features

Of the 6 cases we collected, 4 were adenocarcinomas of the lung, 1 was a squamous cell carcinoma of the lung, and the other was a small cell carcinoma of the lung, as immunohistochemistry was able to help us demonstrate, and immunohistochemical staining of the metastatic lesion and the primary lung lesion were consistent (Table 2). We found that most of the cases of kidney metastasis from lung cancer had poorly differentiated tumors originating from the lung, and only two cases were moderately differentiated, specifically half of the cases had lymph node metastasis. Because some cases were biopsy specimens, the metastases of vasular invasion and lymph nodes metastasis cannot be described in detail (Table 2). The histological differentiation of the tumors did not change significantly after they metastasized to the kidney. Interestingly, our evaluation revealed that the Ki67 of the primary tumor cells was higher than that of the metastatic tumors, suggesting a decreased proliferative capacity of the tumor cells after metastasis, which may be related to the patient’s treatment (Table 2). In lung and kidney biopsy specimens of case 3, the histological morphology of the cells showed the typical features of small cell carcinoma. Loosely adherent hyperchromatic cells with high nuclear/cytoplasmic (N:C) ratio, sparse cytoplasm, nuclear formation, and extrusion artifacts (Fig. 1c,d). Although primary small cell carcinoma of the kidney has been reported [7], preexisting small cell carcinoma of the lung suggests a metastatic process rather than a new primary small cell carcinoma. Wilm’s tumor must be considered in the differential diagnosis, especially in younger patients [8], but this tumor is characterized by very small primitive epithelial cells arranged in rosettes or tubules (blastocysts) and a fibrous/myxoid stroma, which in our csae no features were seen. Histochemistry showed TTF-1 was positive, CgA was paranuclear punctate positive, and the urothelial marker GATA3 was negative (Fig. 1e-g), which well proved that the source of the kidney tumor in this case was lung small cell carcinoma. The cells grow in nests and clusters with obvious nuclear atypia (Fig. 2c,d). Histochemistry showed that P40 and P63 were strongly positive, and the urothelial marker GATA3 was almost negative (Fig. 2e-g), all of the above indicated that was squamous cell carcinoma. We could see that the tumor cells were arranged in adenoids, and the morphology was similar to tumor cells primary in the lung (Fig. 3c,d). Poorly differentiated adenocarcinoma needs to be differentiated from primary sarcomatoid renal cell carcinoma or primary high-grade transitional cell carcinoma (TCC), but spindle cell components were not seen in this case and tumor cells lack the firmness typical of TCC glassy cytoplasm. In particular, histochemistry showed positive for NapsinA and TTF-1, and negative for renal origin marker PAX-8 (Fig. 3e-g) which proved it was lung adenocarcinoma metastases to the kidney.

**Table 2:** Pathological characteristics of the Six Metastases to the Kidney from Primary Lung Cancer Patients

sr No.	Lung	Histological grading	Lymph node metastasis	Vasular invasion	Clinical Staging	IHC	Ki67	Renal	IHC	Histological grading	Vasular invasion	Ki67
1	ACA	P	Y	UN	IVA	CK7+,TTF-1+,NapsinA+, P63-, P40-,Syn-, CgA-,CD56-	80%	ACA	CK(AE1/AE3)+,CK7+, TTF-1+,NapsinA+, PAX-8-,GATA3-	P	Y	60%
2	ACA	M	Y	Y	IIB/ pT1cN1M0	CK7+,TTF-1+,NapsinA+, P63-, P40-,Syn-, CgA-,CD56-	10%	ACA	CK(AE1/AE3)+,TTF-1+, NapsinA+,PAX-8-	P	N	3%
3	Small-cell CA	P	Y	UN	IIIB	TTF-1+,Syn+,CgA+, CD56+,CK7-,NapsinA-, P63-, P40-	90%	Small-cell CA	Syn+, CgA+, TTF-1+,GATA3-	P	UN	60%
4	SCC	P	UN	UN	IIB	P63+,P40+,TTF-1-,CK7-, NapsinA-,Syn-,CgA-,CD56-	40%	SCC	CK(AE1/AE3)+,P63+, P40+,PAX-8-,GATA3-	P	UN	30%
5	ACA	M	UN	UN	IIIA	CK7+,TTF-1+,NapsinA+, P63-, P40-,Syn-, CgA-,CD56-	70%	ACA	CK(AE1/AE3) +, TTF-1 +, NapsinA+, GATA3-	M	UN	40%
6	ACA	M	N	N	IA3 / pT1cN0M0	CK7+,TTF-1+,NapsinA+, P63-, P40-,Syn -, CgA-,CD56-	60%	ACA	CK7+,TTF-1+, NapsinA+,PAX-8-,GATA3-	M	N	20%

ACA, adenocarcinoma; CA, carcinoma; SCC, squamous-cell CA;N,no;Y,yes;UN, unknown;P,poorly differentiated;M, moderately differentiated,CK, cytokeratin; TTF-1, thyroid transcription factor-1; NapsinA,Aspartic proteinase napsin;PAX-8, paired box gene 8;GATA3 ,gata binding protein 3;Syn,synapsin;CgA, chromogranin A

### Treatment and follow-up

Of the six cases we studied, since case 1 was transferred from another hospital, both lung and kidney tumors were found at the time of presentation at our hospital, and a kidney biopsy clarified the metastasis of lung cancer, so he was initially staged for IVA and then underwent laparoscopic radical nephrectomy. Brain metastases developed 12 months after the discovery of renal metastases and died 14 months after the discovery of renal metastases. Case 2 started a right lung lobectomy with a pathological stage of pT1cN1M0 (IIB) ,and underwent laparoscopic partial nephrectomy then receiving chemotherapy and is currently being followed up. Case 3 with initial tage of IIIB received radiotherapy and chemotherapy, developed brain metastases 5 months after the discovery of renal metastases, and died 15 months after the discovery of renal metastases. Case 4 with initial tage of IIB received a combination of chemotherapy and immunotherapy and died 7 months after the discovery of renal metastases. Case 5 with initial tage of IIIA received chemotherapy and immunotherapy and is currently being followed up. Case 6 received a right lung lobectomy with a pathological stage of pT1cN0M0 (IA3) at first and underwent laparoscopic partial nephrectomy then receiving chemotherapy and immunotherapyand is currently being followed up. Case 1 with radical nephrectomy but developed brain metastases later and dead of disease whose survival term was 14 months. Case 2 and case 6 received partial nephrectomy without metastasis at other sites, and their survival term were 55 and 16 months, who are still being followed up. In our limited data, we found that the mean OS was longer in patients who received nephrectomy for single metastasis compared with those who did not undergo surgery. As a whole, the prognosis of lung cancer patients with renal metastases is very poor in terms of prognosis, and the prognosis is even worse in cases presenting with multiple sites of metastases.

## Discussion

We retrospectively reviewed 1555 cases of malignant tumors occurring in the kidney including 9 cases of metastatic tumors between January 2010 to January 2022 in the archives of our department of pathology. Regarding the source of the primary tumor, the most common was lung cancer (0.38%), followed by liver cancer(0.12%), and osteosarcoma (0.064%). Lung cancer can metastasize to any organ, major sites of metastases include brain, bone, and adrenal glands, other organs are involved usually in late stage of the disease. Among different lung cancer types, there are also preferential metastatic sites, such as liver metastases from small cell lung cancer (SCLC) and brain metastases from SCLC or adenocarcinoma [9]. Cases of metastases to the kidney are relatively rare, and are mainly reported in single cases [5]. In current study about renal metastatic cancer, the lung is the most common source of metastases, followed by colorectal, gastric and breast malignancies, and most patients do not have specific urologic symptoms [10]. Interestingly, more than two-thirds of patients develop isolated renal metastases, but most eventually develop metastases in other organs [11, 12]. Considering that the kidney is a highly vascularized organ, metastatic infiltration is likely due to arterial embolization [13].

Distinguishing between primary and secondary tumors of the kidney is essential to guide treatment and prevent unnecessary surgery. However, the clinical presentation of patients with renal metastatic carcinoma may be similar to that of renal cell carcinoma, presenting as a single mass with hematuria. Therefore, the initial diagnosis of renal metastases usually comes from routine imaging, but the final diagnosis needs to rely on pathological diagnosis [2]. Aspiration biopsy is often used to diagnose renal metastases before surgery [14]and in half of the six cases in this study also used. Because of the low degree of differentiation of tumor cells in the primary foci of lung cancer metastasizing to the kidney and the relatively small and atypical morphology of the tissue in the biopsy specimens, they easily overlap with primary renal tumors such as renal cell carcinoma and uremic syndrome. Therefore, it is necessary to take a detailed medical history and make full use of immunohistochemical techniques to help us make a differential diagnosis, which is very important for clinical treatment. Therefore, clinical data, radiological data, histological correlation of the primary tumor and immunohistochemical findings help to determine the correct pathological diagnosis [15].

In these six cases, the patients were all male and the mean age was 62 years, which is consistent with other reports in the literature [16]. In our study, Case 1 with a poorly differentiated adenocarcinoma of the lung, received radiotherapy and chemotherapy, and underwent radical nephrectomy. Later, he developed brain metastases and dead of disease. Case 2 with moderately differentiated adenocarcinoma of the lung received partial nephrectomy, and only chemotherapy was performed, but the follow-up is still underway. Case 3 with lung small cell carcinoma had the shortest time to metastasize to the kidney. After receiving radiotherapy and chemotherapy, then brain metastases occurred, and dead of disease. Case 4 with poorly differentiated squamous cell carcinoma of the lung received radiotherapy and immunotherapy without surgery, and finally dead of disease. Case 5 and Case 6 were both with moderately differentiated adenocarcinomas of the lung, of which case 6 underwent partial nephrectomy, both received radiotherapy and immunotherapy, and are currently being followed up. From the clinicopathological results, we found that the degree of differentiation of tumor cells after metastasis from lung cancer to the kidney did not change significantly, which may be because the degree of differentiation of the primary tumor was mainly low differentiation, but the Ki67 of tumor cells decreased after metastasis, suggesting that the proliferation activity of tumor decreased, which may be related to clinical treatment. Four of these cases were lung adenocarcinomas, which is consistent with the incidence of different histological types of lung cancer that adenocarcinomas are more common than other types. From the clinical data about metastases to the kidney from primary lung cancer, the clinical symptoms of the six patients were not obvious, and only two patients had hematuria, mainly due to imaging findings. Lung squamous cell carcinoma and lung small cell carcinoma metastasized to the kidney in a relatively short time, while the metastatic time of lung adenocarcinoma was related to its degree of differentiation. The worse the differentiation, the faster the metastasis. Three of the patients underwent nephrectomy, one with laparoscopic radical nephrectomy and two with laparoscopic partial nephrectomy. By reviewing the medical history and consulting with the relevant clinicians, we found that all three patients did not have a definitive preoperative biopsy, and were found to have renal metastases after resection by pathological diagnosis. Notably, they had no metastases found at other sites prior to this, and the reasons for surgery in each of the three patients were therapeutic purpose, suspected primary tumor of kidney and poor kidney function.Whether surgical treatment of renal metastases can improve the prognosis of patients remains controversial now. For patients with solitary renal metastases and reported in the literature, nephrectomy can significantly prolong the survival time of patients [2], and we found that the mean OS was longer in patients who received nephrectomy for single metastasis compared with those who did not undergo surgery in our limited data.

The current primary goal of systemic therapy in patients with metastatic NSCLC is to reduce the symptom burden of the cancer and improve survival while aiming to improve quality of life [9]. Platinum-based combination chemotherapy regimens (e.g., carboplatin and paclitaxel or carboplatin and pemetrexed) have been shown to improve survival compared with single-agent chemotherapy, and in some patients, surgery, radiation, or both may be required to treat symptoms [17]. Notably, immunotherapy is one of the major advances in the treatment of advanced tumors in recent years, and non-small cell lung cancer (NSCLC) is one of the cancers that benefits the most from this approach. Currently, the only validated companion diagnostic test for first-line immunotherapy in patients with metastatic NSCLC is the expression of programmed death ligand 1 (PD-L1) in tumor tissue [18]. Small cell lung cancer (SCLC) has different pathological, clinical and molecular features from non-small cell lung cancer. SCLC has a high metastatic potential, resulting in poor clinical prognosis. Concurrent chemoradiotherapy is currently the standard treatment for SCLC, and the Food and Drug Administration has also accelerated approval of nivolumab for the third-line treatment of metastatic SCLC [3]. All patients in this study received chemotherapy, some patients chose to combine radiotherapy and chemotherapy, and some patients received immunotherapy combined with chemotherapy, which may be related to the high expression of PD-L1. It has been reported in the literature that stereotactic body radiation therapy is well tolerated and safe for metastatic renal cancer, and it can provide good symptom relief and early local control [19].

In this study, we showed six cases of lung cancer metastasis to the kidney from different tissue subtypes, and described and analyzed their clinical features, pathological features, treatment methods and prognosis, in order to provide help for the pathological diagnosis and clinical treatment of lung cancer with renal metastasis in the future.

## Data Availability

The raw data supporting the conclusions of this article will be made available by the authors, without undue reservation.

## References

[1] Patel TV, Cornell L, Wolf M (2008). Renal metastases. KIDNEY INT.

[2] Chen J, Qi N, Zhu S. Metastases to the Kidney: An Analysis of 35 Cases and a Review of Literature. FRONT ONCOL (2021) 10. 10.3389/fonc.2020.632221 PMC7934622 33680955

[3] Semenova EA, Nagel R, Berns A (2015). Origins, genetic landscape, and emerging therapies of small cell lung cancer. Genes Dev.

[4] Herbst RS, Morgensztern D, Boshoff C (2018). The biology and management of non-small cell lung cancer. Nature.

[5] Numan L, Asif S, Abughanimeh O. Isolated Renal Metastasis from Primary Lung Squamous Cell Carcinoma with Synchronous Small Cell Lung Cancer. *Cureus*. 10.7759/cureus.4891 PMC6689493 31423371

[6] Riihimaki M, Hemminki A, Fallah M, Thomsen H, Sundquist K, Sundquist J (2014). Metastatic sites and survival in lung cancer. Lung Cancer.

[7] Capella C, Eusebi V, Rosai J (1984). Primary oat cell carcinoma of the kidney. AM J SURG PATHOL.

[8] Quijano G, Drut R (1989). Cytologic characteristics of Wilms’ tumors in fine needle aspirates. A study of ten cases. Acta Cytol.

[9] Popper HH (2016). Progression and metastasis of lung cancer. CANCER METAST REV.

[10] Zhou C, Urbauer DL, Fellman BM, Tamboli P, Zhang M, Matin SF (2016). Metastases to the kidney: a comprehensive analysis of 151 patients from a tertiary referral centre. BJU INT.

[11] Hietala SO, Wahlqvist L (1982). Metastatic tumors to the kidney. A postmortem, radiologic and clinical investigation. Acta Radiol Diagn (Stockh).

[12] Patel U, Ramachandran N, Halls J, Parthipun A, Slide C (2011). Synchronous renal masses in patients with a nonrenal malignancy: incidence of metastasis to the kidney versus primary renal neoplasia and differentiating features on CT. AJR Am J Roentgenol.

[13] Khan F, Mahmalji W, Sriprasad S, Madaan S. Prostate cancer with metastases to the kidney: a rare manifestation of a common disease. *BMJ Case Rep* (2013) 2013. 10.1136/bcr-2012-008388 PMC3762516 23907962

[14] Anik Sahni VREVIEW (2009). Biopsy of renal masses: when and why. Cancer Imaging.

[15] Choyke PL, White EM, Zeman RK, Jaffe MH, Clark LR (1987). Renal metastases: clinicopathologic and radiologic correlation. Radiology.

[16] Chen J, Qi N, Wang H, Wang Z, He Y, Zhu S. Second Primary Renal Cell Carcinoma With Nonrenal Malignancies: An Analysis of 118 Cases and a Review of Literature. FRONT ONCOL (2021) 11. 10.3389/fonc.2021.780130 PMC8656157 34900734

[17] Arbour KC, Riely GJ (2019). Systemic therapy for locally Advanced and Metastatic non–small cell Lung Cancer. JAMA.

[18] Brozos-Vázquez EM, Díaz-Peña R, García-González J, León-Mateos L, Mondelo-Macía P, Peña-Chilet M (2021). Immunotherapy in nonsmall-cell lung cancer: current status and future prospects for liquid biopsy. Cancer Immunol Immunother.

[19] Verma V, Simone NCB (2017). Stereotactic body radiation therapy for metastases to the kidney in patients with non-small cell lung cancer: a new treatment paradigm for durable palliation. Annals of palliative medicine.

